# Role of ferroptosis in effects of anesthetics on multiple organ diseases: A literature review

**DOI:** 10.1016/j.heliyon.2023.e20405

**Published:** 2023-09-23

**Authors:** Gulibositan Abudurousuli, Siyang Xu, Jinxing Che, Xiahao Ding, Bo Gui, Linjia Zhu

**Affiliations:** a Department of Anesthesiology and Perioperative Medicine, The First Affiliated Hospital with Nanjing Medical University, Nanjing, China; b Department of Anesthesiology, Jiangsu Province Official Hospital, Nanjing, China; c Department of Anesthesiology, The Huai'an Maternity and Child Healthcare Hospital, Huai'an, China

**Keywords:** Ferroptosis, Anesthetics, Multiple organ diseases, Glutathione peroxidase 4

## Abstract

Anesthesiologists are often faced with patients combined with a series of organ injuries, such as acute lung injury, myocardial ischemia-reperfusion injury, and neurodegenerative diseases. With the in-depth study of these diseases, we are more aware of the choice and rational use of anesthetics for the prognosis of these patients. Ferroptosis is a new type of programmed cell death. This unique pattern of cell death, driven by an imbalance between oxides and antioxidants, is regulated by multiple cellular metabolic events, including redox homeostasis, iron handling, mitochondrial activity, and lipids peroxidation. Numerous studies confirmed that anesthetics modulate ferroptosis by interfering its machineries such as cystine-import-glutathione-glutathione peroxidase 4 axis, Heme oxygenase 1, nuclear factor erythroid 2-related factor 2, and iron homeostasis system. In this literature review, we systemically illustrated possible involvement of ferroptosis in effects of anesthetics and adjuvant drugs on multiple organ diseases, hoping our work may serve as a basis for further studies on regulating ferroptosis through anesthetics related pharmacological modulation and promoting the rational use of anesthetics.

## Abbreviations

ACSL4: Acyl-CoA synthetase long-chain family member 4
DMT1: divalent metal ion-binding protein 1
GPX4: glutathione peroxidase 4
GSH: glutathione
4-HNE: 4-hydroxy-2-nonenal
HO-1: Heme oxygenase 1
LPCAT3: Lys phosphatidylcholine acyltransferase 3
L-ROS: lipid-reactive oxygen species
MDA: malondialdehyde
NF-κB: nuclear factor-kappa B
Nrf2: nuclear factor erythroid-2 related factor 2
PUFAs: polyunsaturated fatty acids
ROS: reactive oxygen species
SLC7A11: Beclin1-solute carrier family 7member 11
TFRC: transferrin receptor

## 1 Introduction

Ferroptosis is a distinct form of programmed cell death, which results from a series of essential metabolic process disorders, leading to iron-dependent, lipid-reactive oxygen species (L-ROS)-mediated cell destruction. Ferroptosis can be induced by inhibiting glutathione peroxidase 4 (GPX4), which produces glutathione (GSH) [1] and depleting polyunsaturated fatty acids (PUFAs) on the plasma membrane [2]. GSH depletion triggers ferroptosis and toxic L-ROS accumulation. Meanwhile, antioxidant depletion leads to excessive levels of ROS, which damage cellular DNA, lipids, and proteins, and result in cell death [3]. The twelve-pass transmembrane transporter system xc^−^ (consisting of Beclin1-solute carrier family 7 member 11 (SLC7A11) complex) controls cystine glutamate anti-transportation. Inhibition of system xc− may initiate erastin-induced ferroptosis [4,5]. Thus, ferroptotic cell death is the downstream of system xc^−^ inhibition or GPX4 inactivation. Iron is essential for the occurrence of ferroptosis. Its utilization mainly occurs in mitochondria, which are the major site for the biogenesis of iron–sulfur clusters and perform various metabolic events, such as heme synthesis. However, the mechanism of mitochondrial iron transport is still unclear. To sum up, the main mechanisms of ferroptosis includes iron metabolism, amino acid metabolism and lipid metabolism. On top of that, other signal pathways and genes also take part in the regulation of ferroptosis. Therefore, modulating ferroptosis process to achieve desirable therapeutic outcomes for the patients with multiple organ diseases is of great interest to the clinicians.

Ferroptosis is influenced by several genes that are involved in lipid and amino acid metabolism. Genetic screening results showed that acyl-CoA synthetase long-chain family member 4 (ACSL4), Lys phosphatidylcholine acyltransferase 3 (LPCAT3), cytochrome P450 oxidoreductase, and Kelch-like ECH-associated protein 1 regulate the ferroptosis process [6]. Silencing the gene related to transferrin receptor (TFRC) could disrupt the cellular uptake of transferrin-iron complexes and inhibit ferroptosis. Conversely, upregulation of TFRC and ACSL4 expression enhances ferroptosis [7]. The prominent tumor suppressor gene P53 has a dual role in ferroptosis. On one hand, it induces the expression of several antioxidant genes that are expected to reduce ROS accumulation and prevent ferroptosis. On the other hand, P53 upregulation suppresses SLC7A11 expression and makes cells more susceptible to ferroptosis [8]. Nrf2 is a key regulator of ferroptosis-related genes, some diseases can be relieved by targeting on the Nrf2 activation which could lead ferroptosis to a halt.

Ferrostatin-1 is a ferroptosis inhibitor that can suppress iron-dependent lipid peroxidation and prevent the development and progression of various organ diseases, such as acute lung injury and renal and myocardial ischemia–reperfusion injuries. This suggests that ferroptosis is closely related to major organ dysfunction [[9], [10], [11]]. Increasing evidence has shown that anesthetics and adjuvant drugs can either increase or decrease oxidative stress by affecting the ferroptosis process. In this review, we summarized the effects of anesthetics and adjuvant drugs on multiple organ diseases and the possible role of ferroptosis [12]. We also provide a detailed explanation of how ferroptosis may be involved in the effects of glucocorticoids and mechanical ventilation on major organ injuries. Look back on the past, achievements and progresses made in anesthesia have brought huge benefits to human health. In recent years, with the great efforts made by scientists to deal with multiple organ diseases, severe trauma and perioperative disorders by regulating ferroptosis, no wonder anesthesiologists begin to pay attention to the mechanism of ferroptosis. Considering that clinical anesthesiologists often encounter patients with multiple organ diseases, this literature review may be very appealing. It may serve as a basis for further clinical research on regulating ferroptosis and promoting the rational use of anesthetics.

## 2 Respiratory system

Ferroptosis is a type of cell death that may play a crucial role in the onset and progression of acute lung injury/acute respiratory distress syndrome (ALI/ARDS), characterized by extensive destruction of pulmonary vascular and alveolar epithelial cells [13,14]. The discovery of ferroptosis also expanded the use of anesthetics for preventing ALI/ARDS. Dexmedetomidine and sevoflurane might inhibit cellular ferroptosis and alleviate vascular endothelial cells damage by enhancing GPX4 and GSH activities and improving mitochondrial morphology. Glucocorticoids combined with ferroptosis-inducing agents could induce eosinophil death and reduce allergic airway inflammation, which may supply a fresh idea for clinical asthma therapy.

### 2.1 Dexmedetomidine

Dexmedetomidine is a highly selective α_2_-adrenergic receptor agonist with sedative and mild analgesic properties. It exerts anti-inflammatory, antioxidant, and anti-apoptotic effects in many organ diseases, including sepsis in the lungs [[15], [16], [17]]. In a sepsis model induced by cecal ligation and puncture, dexmedetomidine upregulates the expression of glucose-6-phosphate dehydrogenase by nuclear factor erythroid 2-related factor 2 (Nrf2) and activates the pentose phosphate pathway, thereby increasing GPX4 and GSH activities, boosting free radical scavenging, and inhibiting lipid peroxidation and ferroptosis [18]. These results revealed that dexmedetomidine infusion may be an effective approach to treating septic patients. Ferroptosis is involved in the progression of sepsis, which damages the cellular mitochondrial cristae and makes them swollen, sparse, and even vacuolated [9]. Dexmedetomidine counteracts sepsis-induced excessive mitochondrial fission to improve mitochondrial function, reduce ROS production, and protect vascular endothelial cells by impeding ferroptosis [18]. Convincingly, dexmedetomidine is a promising therapeutic agent for sepsis-induced vascular leakage and organ dysfunction by targeting ferroptosis.

### 2.2 Sevoflurane

Sevoflurane, a popular volatile anesthetic, is the first-line clinical option for anesthesiologists partly due to its protective effects against ALI. It mitigates the worsening effects of ferroptosis inducer Fe-citrate on lipopolysaccharide (LPS)-induced ALI in a mice model [19]. In the lung tissue of ALI mice model, sevoflurane inhibited LPS-induced ferroptosis by reducing the accumulation of malondialdehyde (MDA) and Fe^2+^ and increasing the activities of Heme oxygenase 1 (HO-1), GSH, and GPX4 [19]. HO-1 breaks down heme into nitric oxide, biliverdin, and iron to provide an effective antioxidant defense against cellular stress, which is related to ferroptosis [20]. Pre-and post-conditioning with sevoflurane might protect against ALI by upregulating HO-1 expression [21,22]. Taken together, these results showed that ferroptosis contributes to the prognosis of ALI, and sevoflurane inhibits ferroptosis by upregulating HO-1 expression to ameliorate LPS-induced ALI, which provides novel insights for the clinical application of sevoflurane in ALI therapy.

### 2.3 Glucocorticoids

Glucocorticoids are potent anti-inflammatory agents. They can regulate inflammation-regulate lung injury and thus delay progression to respiratory failure and death [23,24]. In an LPS-induced mouse model of ALI, dexamethasone administration downregulated the expression of interleukin (IL)-6, tumor necrosis factor (TNF)-α, myeloperoxidase, and MDA and upregulated that of GSH by activating the nuclear factor-kappa B (NF-κB) P65 pathway [25]. However, dexamethasone might sensitize cells to ferroptosis. GSH levels were dramatically decreased due to upregulation of its regulator protein dipeptidase-1 (DPEP1) in a glucocorticoid receptor-dependent manner [26]. Eosinophils, a key feature of allergic asthma, are associated with the occurrence of ferroptosis owing to their abundance of iron. Promoting ferroptosis in eosinophil may effectively attenuate allergic airway inflammation and its related diseases. Ferroptosis-inducing agents (FINs) induce noncanonical ferroptosis in eosinophils, and dexamethasone combined with FINs could be a novel synergistic therapy for eosinophil death and allergic airway inflammation [27]. Moreover, ferroptosis was implicated in dexamethasone-induced toxicity on zebrafish larvae, which provides strong evidence for the harmful effects of glucocorticoids on aquatic organisms [28]. The dose and frequency dependent effects of glucocorticoids on ferroptosis need to be further elucidated. Glucocorticoid-induced osteonecrosis of femoral head (GIONFH) is a severe complication of long-term administration of glucocorticoids. A total of 27 ferroptosis-related differentially expressed genes involved in the pathological process of GIONFH were identified via bioinformatics analysis. Toll-like receptor 4 (TLR4), thioredoxin-interacting protein, and MAP3K5 might serve as potential biomarkers and drug targets for GIONFH [29]. Together, we upgraded a previously incomplete cognition of the mechanism of glucocorticoid-mediated sensitization to ferroptosis and identified FINs as a promising therapeutic strategy for allergic airway inflammation.

## 3 Central nerve system

Iron is essential for brain development and multiple neurofunctions [30]. However, overloaded iron capacity causes neurotoxicity, neurodegenerative diseases, and cognitive impairments via inflammatory response and oxidative stress-induced signaling pathways [[31], [32], [33], [34]]. In addition, iron accumulation in the brain can initiate the Fenton reaction and produce ROS, which further induce cell apoptosis and neuro-dysfunction. These data indicate that ferroptosis accounts for neuronal death in various pathological conditions. There is increasing evidence that clinical doses of propofol and dexmedetomidine protect neurons by lowering the levels of cellular iron, lipid peroxides, and reactive oxygen species. Furthermore, ferroptosis may be involved in the effects of inhaled anesthetics on neurodegenerative diseases, such as postoperative cognitive dysfunction. Treatment targeting the ferroptosis process might significantly improve learning and memory abilities. These findings may have important implications for improving anesthesia management and preventing perioperative complications.

### 3.1 Propofol

The mechanism of how propofol chelates iron to protect against nerve injury is still unclear, but some potential pathways have been identified. Propofol protects cultured brain cells from iron ion-induced death, and its combination with a vitamin E analog prolongs its protection against oxidative stress [35]. When cellular iron levels exceed the normal requirement, the expression of the iron storage protein ferritin increases, whereas that of iron import transferrin receptor 1 (TfR1) decreases. In addition to TfR1, divalent metal ion-binding protein (DMT1) and ferroportin1 (Fpn1) play important roles in regulating cellular iron homeostasis. In the human neuroblastoma cell line SH-SY5Y, a low concentration of propofol (5 μM) can significantly prevent ferric citrate-induced apoptosis by inhibiting oxidative stress and iron accumulation, downregulating the expression of ferritin, and upregulating the expression of TfR1 and Fpn1 but not the DMT1 pathway. Meanwhile, propofol suppresses iron overload-induced inflammatory response by inhibiting IL-6 and COX2 expression and activating Janus Kinase (JAK)/Signal Transducers and Activators of Transcription (STAT) 3 signaling [36]. Hence, propofol may protect against iron overload-related nerve cell injury through regulating iron metabolism. In mouse brains model, propofol also protects against cerebral ischemia reperfusion injury (CIRI)-induced ferroptosis partly by activating the Nrf2/GPX4 signaling pathway [37]. However, treatment of hippocampal neurons with 100 μM of propofol decreases the hippocampal neuron viability and causes long-term learning and cognitive dysfunction. Neurotoxic effects of propofol might be attributed to decreased superoxide dismutase (SOD) content and increased ROS and Fe^2+^ content and neuronal apoptosis as well as up-expression of DMTI and TfR1 proteins and down-expression of GPX4, SLC7A11 and FPN1. Furthermore, hypoxic preconditioning reverses these noxious effects, inhibits propofol-induced ferroptosis and neurotoxicity [38]. These controversial results may be attributed to the variations in the animal experimental models. Thus, whether propofol has a beneficial or detrimental effect on central nervous system injury needs to be further confirmed.

### 3.2 Dexmedetomidine

Previous studies have demonstrated the neuroprotective effect of dexmedetomidine in models of hypoxic/ischemic and hemorrhagic brain injury [39,40]. Dexmedetomidine has an antioxidative effect on the PC12 cell line derived from pheochromocytoma partly by preventing intracellular iron accumulation caused by the oxidative stress inducer *tert*-butyl hydroperoxide [41]. In the SK-N-SH nerve cell model, treatment with 5 μM of dexmedetomidine alone decreases the mRNA levels of TfR1, iron regulatory protein 1 (IRP1), and labile Fe^2+^. Dexmedetomidine may regulate iron metabolism by controlling iron importers and exporters via the phosphorylation of c-Jun N-terminal kinase/SP-1 and signal transducer and activator of transcription 4/SP-1 signaling. Thus, dexmedetomidine might protect SK-N-SH nerve cells from oxidative injury by maintaining iron homeostasis [42]. Low concentration of dexmedetomidine inhibits ferroptosis, whereas high concentration of dexmedetomidine regulates apoptosis. Consistently, in an iron overload model of SH-SY5Y cell, 20 μM of dexmedetomidine significantly attenuates ROS accumulation and inhibits apoptosis by inhibiting the NF-κB signaling pathway but fails to reduce iron entry into cells [43]. All in all, these interesting results might promote the clinical researches for exploring efficacy and possible mechanism of dexmedetomidine in treating brain injuries.

### 3.3 Ketamine

Ketamine is an anesthetic drug that has hypnotic and analgesic effects. It may cause neurotoxicity by affecting an iron transport signaling pathway. This pathway involves the upregulation of N-methyl-d-aspartate receptor (NMDAR), which increases iron influx through DMT1, stimulates iron release from lysosomes, and leads to iron-induced cell damage. In primary hippocampal neurons, young rats and aged mice models, ketamine decreases the expression of IRP2 and TfR1, increases the expression of ferritin, triggers iron-mediated ferroptosis, activates DMT1-mediated NMDAR-RASD1 signaling, and eventually causes mitochondrial dysfunction and cognitive deficits [44]. Ketamine significantly increased level of Mfrn1, a protein located in the inner mitochondrial membrane, in cultured hippocampal neurons [44]. Major depressive disorder (MDD) is a burdensome condition with few treatment options, and traditional antidepressants are characterized by slow onset. However, sub-anesthetic ketamine exerts rapid-onset effects for the treatment of MDD by inhibiting ferroptosis in the nuclear complex with increased FTH1 and GPX4 levels and decreased TfR1 levels [45]. Under electron microscopy, ketamine was also found to restrain ferroptosis, increase FTH1 and GPX4 levels, and decrease TfR1 levels [45]. These results suggest that some non-narcotic effects of ketamine are possibly based on development of ferroptosis process.

### 3.4 Sevoflurane

Sevoflurane exposure may result in cognitive deficits in neonatal animals. The potential mechanism of sevoflurane-induced neurotoxicity needs to be deeply investigated. Excess iron leads to neurotoxicity and cognitive impairment. In neurons, sevoflurane induced iron regulatory protein (IRP2)- and TfR1-mediated iron overload and then led to ferroptosis [44]. In a model of sevoflurane-aggravated brain injury of young rats, TFRC levels and ACSL4 levels were elevated after sevoflurane administration, suggesting that sevoflurane aggravates the extent of ferroptosis [46]. Sevoflurane administration elicited mitochondrial dysfunction and iron dyshomeostasis, and eventually resulted in cognitive impairments in neonatal mice, while chelating neurotoxic iron could effectively improve sevoflurane-induced cognitive deficits [47]. Sevoflurane could cause neurogenesis abnormality and ferroptosis but not apoptosis in embryonic prefrontal cortex [48]. In the 15-month-old mice, sevoflurane induced iron overload, which led to impaired mitochondrial function and disordered glucose metabolism. This cross-dysfunction of iron and glucose metabolism triggers apoptosis in the cortex and hippocampus via the Bcl2/Bax pathway [49]. Treatment of sevoflurane increased Fe^2+^ level and decreased SLC7A11 and GPX4 mRNA levels in SH-SY5Y cells. Sevoflurane also increased the expression of ACSL4, which contributed to ferroptotic neuronal death in SH-SY5Y cells via the 5ʹ AMP-activated protein kinase/mammalian target of rapamycin pathway. Downregulation of ACSL4 restrained sevoflurane-induced ferroptotic cell death via AMPK/mTOR signaling, providing a basis for developing an approach to alleviate sevoflurane-induced postoperative cognitive dysfunction [50,51]. Echinatin has anti-inflammatory and antioxidant activity. It activated Nrf2 in hippocampal neurons and alleviated sevoflurane-induced neurotoxicity and cognitive deficits by mitigation of ferroptosis and oxidative stress in the aged rats [46]. Sevoflurane treatment also induced ferroptosis in glioma cells. Sevoflurane was reported to increase ROS levels and Fe^2+^ concentration, downregulate the GPX4 protein expression, and upregulate transferrin, ferritin, and Beclin-1 in a dose-dependent manner by activating the ATF4-CHAC1 pathway in the two kinds of glioma cells [52]. However, sevoflurane can also exert protective effects by counteracting ferroptosis. In a middle cerebral artery occlusion model, sevoflurane post-conditioning reduces intracellular iron accumulation, inhibits ferroptosis, and alleviates neurological deficits and cerebral infarction after ischemia/reperfusion injury (IRI). In this model, it was confirmed that the specificity protein 1 (SP1)/ASCL4 axis was inhibited with administration of sevoflurane. SP1 shaped ferroptosis sensitivity via either transcription-dependent or transcription-independent manners. To be concrete, SPI upregulates ACSL4 expression by binding to the ACSL4 promoter region and thus ACSL4 is identified as a direct target gene for SPI [53]. This finding suggests a novel perspective for cerebral protective treatment against cerebral IRI and indicates a potential therapeutic approach for various cerebral diseases.

### 3.5 Isoflurane

Isoflurane is another commonly used inhalational anesthetic. Isoflurane induced the ferroptosis in a dose-dependent and time-dependent manner in hippocampus. Isoflurane exposure (1.5% isoflurane exposure for 6 h) increased the activity of cytochrome *c* oxidase/Complex IV and contributed to cysteine deprivation-induced ferroptosis. More importantly, isoflurane-induced ferroptosis could be rescued by both ferroptosis inhibitor and mitochondria activator, which might be the effective therapeutic target against isoflurane-induced learning and memory impairment [54]. A previous study using an *in vitro* embryonic mouse model demonstrated that ferroptosis is a central mechanism contributing to isoflurane neurotoxicity and associated with decreased transcription and protein expression of the lipid repair enzyme GPX4. Exposure to 2% isoflurane for 6 h increased ROS generation, disrupted mitochondrial membrane potential, and caused cell death [55]. Ferroptosis inhibitors ferrostatin-1 and deferoxamine mesylate effectively maintained the viability of SH-SY5Y neuroblastoma cells, which were exposed to a high concentration of isoflurane for 24 h. Isoflurane upregulated Beclin1 phosphorylation, followed by the malformation of SLC7A11, which affected the activity of cystine/glutamate antiporter and further regulated ferroptotic cell death [56]. These findings indicate that isoflurane-induced toxicity is modulated by targeting on Beclin1. Beclin1 inhibits the glutamate exchange activity of system xc^−^ and eventually prevent the process of ferroptosis. Therefore, inhibiting ferroptosis process might contribute to alleviate isoflurane-induced neurotoxicity.

## 4 Cardiovascular system

Ferroptosis is involved in doxorubicin- and IRI-induced cardiac injury through the Nrf2/HO-1 axis [57,58]. ROS increases the mitochondrial iron content in cardiomyocytes and results in mitochondrial membrane damage. The accumulation of iron in the serum and heart tissue is independent of the classic hepcidin–ferroportin iron regulatory axis. Through HO-1, liable iron degrades from heme to free iron, iron instability is upregulated and then the plasma iron overload induces ferroptosis in cardiovascular system [57]. Pharmacological inhibition of ferroptosis regulating routines or iron chelation therapy can prevent cardiomyopathy. Rational use of dexmedetomidine, propofol, etomidate, and sevoflurane can reduce the degree of ferroptosis and protect cardiac injury by both the increases in the activities of SOD, GSH, and GPX4 and the decrease in the expression of iron. These findings highlight the potential value of ferroptosis-targeted treatment in ischemic heart disease.

### 4.1 Dexmedetomidine

Dexmedetomidine has a protective effect during IRI in the cardiovascular system [59]. This finding has stimulated further studies to explore its underlying mechanism. The Trx1-dependent Akt pathway and the C/EBP-homologous protein signaling pathway are crucial in the cardioprotective effect of dexmedetomidine [[59], [60], [61]]. Recent studies have suggested that dexmedetomidine alleviates sepsis-induced myocardial cellular injury by attenuating sepsis-induced HO-1 overexpression and increased iron concentration and reducing ferroptosis via enhancing GPX4 [17]. Similar outcomes also apply to IRI model. Dexmedetomidine post-conditioning enhanced the expression of SLC7A11 and GPX4 signaling pathway, inhibited ferroptosis, thereby prevented cardiac IRI [62]. Dexmedetomidine protects heart against myocardial ischemia/reperfusion injury (MIRI)-induced ferroptosis via activation of Nrf2 in models of Sprague-Dawley rats and H9c2 cells [63]. As a redox-sensitive transcription factor, Nrf2 could bind to α_2_-AR. Therefore, dexmedetomidine-induced α_2_-AR activation may be responsible for upregulation of Nrf2. Dexmedetomidine also protected cardiomyocytes from IRI partially by activating GPX4 through the cAMP/protein kinase A (PKA)/element-binding protein (CREB) pathway in models of H9c2 cells [64].

### 4.2 Propofol

P53 promotes fatal lipid ROS accumulation and induces ferroptosis in cardiomyocytes. It is upregulated in IR-induced myocardial injury, and AKT plays a pivotal role in p53 degradation [65,66]. Using an IRI rat model, a recent study has shown that propofol administration inhibits myocardial ferroptosis by activating the AKT/P53 signaling pathway, reducing iron level, and increasing the activities of anti-ferroptosis enzymes (ferritin heavy chain 1, cysteine/glutamate transporter, and GPX4) and antioxidant enzymes in H9c2 cells [67]. These findings confirmed the protective effects of propofol on ferroptosis process during myocardial diseases to a certain extent, which warrants further investigation.

### 4.3 Etomidate

Etomidate is an excellent anesthetic that is widely used because of its myocardial protective effects [68]. In a MIRI rat model, this anesthetic decreased the secretion of inflammatory factors (IL-6, IL-1β, and TNF-α) and inhibited IRI-induced ferroptosis in the myocardium by inducing the increases in the activities of superoxide dismutase (SOD), GSH, and GPX4 and the reduction in the expression of iron and ACSL4 through upregulation of Nrf2 and HO-1 protein expression [69]. These results shed light on the potential treatment of MIRI. Further clinical research should be conducted to reveal the link between etomidate and iron-dependent cell death.

### 4.4 Sevoflurane

Sevoflurane inhalation have been announced that can far outweigh its positive impact on myocardial cell [70]. Ferroportin 1 and mitochondrial ferritin were considerably up-regulated by sevoflurane, while iron regulatory protein 1, DMT1, and Trf1 were significantly down-regulated in IRI cells. Additionally, sevoflurane substantially reduced the levels of total Fe, Fe^2+^, reactive oxygen species, malondialdehyde, and 4-hydroxynonenal (4HNE) in IRI cells [71]. In other words, sevoflurane relieves IRI-induced cardiomyocyte injury by regulating iron homeostasis and ferroptosis, which provides a theoretical basis for treating the surgical patients with IRI cardiovascular diseases under sevoflurane anesthesia.

## 5 Mechanical ventilation and Urinary system injuries

One of the most notable features of ferroptotic cells observed under electron microscopy is the change of mitochondrial morphology. Mitochondrial injury is associated with ventilator-induced kidney injury because mechanical ventilation can change renal blood flow, decrease oxygen delivery and increase oxygen consumption [72]. An imbalance between oxygen delivery and utilization is expected to induce profound renal mitochondrial injury, which may lead to reduced urine output and creatinine elevation [73]. In an IRI rat model, prolonged mechanical ventilation promoted ferroptosis and eventually resulted in kidney injury, as evidenced by creatinine elevation and oliguria or anuria. In particular, it could significantly decrease the levels of GPX4, lipid peroxidation marker 4HNE, and SOD2 in a time-dependent manner. Compared with 4 h of mechanical ventilation or IRI alone, 12 h of mechanical ventilation can decrease GPX4 level in a time-dependent manner, accompanied by elevated 4HNE level and reduced SOD2 level. Prolonged mechanical ventilation exacerbated renal function failure, and the mechanism may be associated with IRI-induced ferroptosis [74]. In addition, dexmedetomidine administration might mitigate tissue damage, inhibit ferroptosis, and downregulate inflammation response following renal IRI, which were associated with the suppression of ACSL4 [75].

## 6 Conclusions

**Fig. 1:**
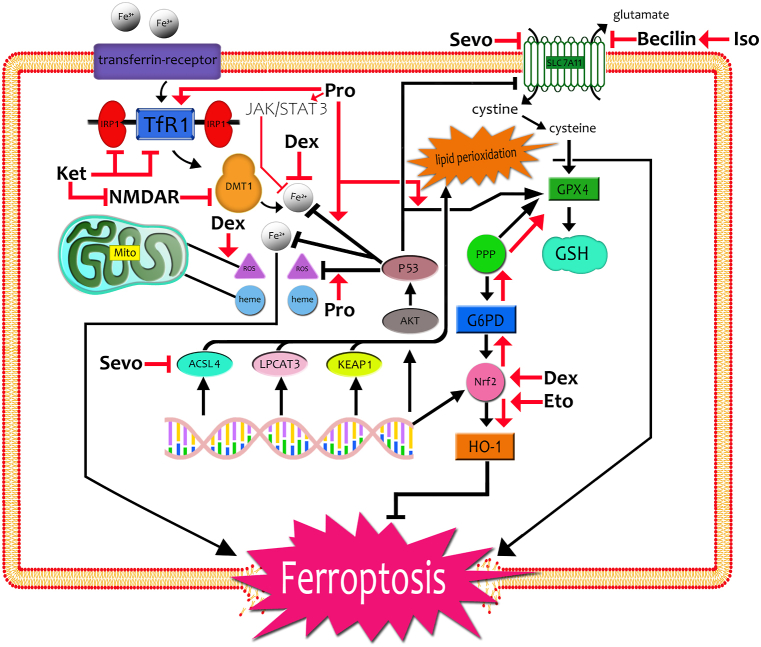
Mechanism of anesthetics and adjuvant drugs affecting the process of cellular ferroptosis. Dex, dexmedetomidine; Eto, etomidate; Sevo, sevoflurane; Pro, propofol; Ket, ketamine; Iso, isoflurane; HO-1, Heme oxygenase 1; Nrf2, nuclear factor erythroid 2-related factor 2; G6PD, glucose-6-phosphate dehydrogenase; PPP, pentose phosphate pathway; GPX4, glutathione peroxidase 4; GSH, glutathione; ACSL4, acyl-CoA synthetase long-chain family member 4; LPCAT3, lysophosphatidylcholine acyltransferase 3; KEAP1, Kelch-like ECH-associated protein 1; Akt, serine/threonine kinase; SLC7A11, Beclin1-solute carrier family 7member 11; NMDAR, *N*-methyl-*d*-aspartate receptor; Mito, mitochondria; ROS, lipid-reactive oxygen species; DMT1, divalent metal ion-binding protein; IRP1, iron regulatory protein 1; TfR1, transferrin receptor1.

Ferroptosis plays remarkable roles in the effects of anesthetics and adjuvant drugs on multiple organ diseases. Depending on the target, anesthetics react differently to ferroptosis in each system (see Fig. 1, Table 1). Inhalational anesthetics might show protective effect against the respiratory system injuries by inhibiting the ferroptosis process and increasing the activities of Heme oxygenase 1 (HO-1), GSH, and GPX4. Meanwhile, intravenous anesthetics, such as dexmedetomidine and etomidate, could benefit patients suffered from myocardial ischemia-reperfusion injury by interfering the process of ferroptosis. However, inhalation anesthetics and ketamine were found to aggravate nerve system injuries partially by ferroptosis in a dose depend manner. Therefore, the role of ferroptosis in major organ injuries and its regulatory way needs to be deeply explored. There has been an exponential increase in the number of studies related to ferroptosis. These findings may serve as the basis for further studies on regulating ferroptosis and promoting the rational use of anesthetics and adjuvant drugs.

**Table 1:** Changes in markers of ferroptosis and their possible mechanism in effects of anesthetics and adjuvant drugs on multiple organ diseases.

System	Drugs/methods	Mechanism	References
Respiratory system	dexmedetomidine	↑: GPX4, GSH, Nrf2 ↓: mitochondrial fission	She et al., 2021[18]
	sevoflurane	↑: GSH, GPX4, SLC7A11, Nrf2, HO-1 ↓: MDA	Dong et al., 2020[20]
		↑: HO-1	Hooper, 2020[21]
			Ngamsri et al., 2022[22]
		↑: HO-1, GSH, GPX4 ↓: MDA, Fe^2+^	Liu et al., 2021[54]
	glucocorticoids	↑: GSH ↓: IL-6, TNF-α, MDA, NF-κB	Wu et al., 2020[27]
		↑: DPEP1 ↓: GSH	Von Massenhausen et al., 2022
Central nerve system	propofol	↑: TFR1, FPN1, JAK, STAT3 ↓: IL-6, COX2, ferritin	Zhang et al., 2019[36]
		↑: Nrf2, GPX4 ↓: LPO	Fan et al., 2022[58]
		↑: ROS, Fe^2+^, MDA, DMT1, TFR ↓: GPX4, SLC7A11, FPN1	Chen et al., 2023[29]
	dexmedetomidine	↑: c-JNT kinase/SP-1 ↓: TFR1, IRP1, Fe (II)	Qiu et al., 2020[42]
		↑: NF-κB ↓: ROS, IL-1β, IL-6, TNF-α	Hu et al., 2019[43]
	ketamine	↑: ferritin, MDA, ROS, LDH, NMDAR-RASD1, DMT1, Mfrn1 ↓: IRP2, TFR1, SOD2, GSH	Wu et al., 2020[44] Zhang et al., 2022[47]
		↑: FTH1, GPX4 ↓: TFR1	Zhang et al., 2022[47]
	sevoflurane	↑: Fe^2+^, AMPK, mTOR ↓: SLC7A11, GPX4	Cheng et al., 2021[50]
		↑: MIB2 ↓: GPX4	Zhao et al., 2021[51]
		↑: ROS ↓: AKT, TFR1, Na+/K + ATPase, Bcl2/Bax	Ge et al., 2021[49]
		↑: ROS, TFR1 ↓: Nrf2, GPX4	Song et al., 2022[48]
		↑: ROS, MDA, ACSL4, COX2 ↓: FTH1, ATP, GSH, mPTP, OCR	P. Zhang et al., 2022[47]
		↑: ACSL4, NSE, Fe^2+^, TFR, ROS	Yu et al., 2022[62]
		↑: LDH, ROS, MDA, Ferritin, Nrf2 ↓: GSH, GPX4	Xu et al., 2022[52]
		↑: transferrin, ferritin, Fe, ROS, Beclin-1, ATF4-CHAC1 ↓: GPX4	Y. Xu et al., 2022[52]
		↑: MDA, iron, ferritin protein ↓: GSH, GPX4	Xu et al., 2022[52]
	Isoflurane	↑: MDA, 4-HNE ↓: GSH, SLC7A11	Liu, P. et al., 2021[54]
		↑: ROS ↓: GPX4	Xia et al., 2018
		↑: Beclin1 ↓: SLC7A11, GSH, GPX4	Liu et al., 2019[43]
Cardiovascular System	dexmedetomidine	↑: GPX4, SOD, GSH ↓: MDA, IL-6, HO-1	Wang et al., 2020[17]
		↑: SLC7A11, GPX4	Yu et al., 2022[62]
		↓: Fe2+, ROS, MDA ↑: Nrf2, SLC7A11, AMPK, GSK-3β	Wang et al., 2022[63]
		↑: GPX4, cAMP, PKA, CREB	Ma et al., 2023[64]
	propofol	↑: GPX4, GSH, FTH1, AKT, P53 ↓: ROS, Fe+	Li et al., 2022[40]
	etomidate	↑: SOD, GSH, GPX4, Nrf2, HO-1 ↓: IL-6, MDA, ACSL4	Lv et al., 2021[69]
	sevoflurane	↑: Ferroportin1, ferritin ↓: IRP1, DMT1, TFR1, Fe, Fe2+, MDA	Sheng et al., 2023[71]
Urinary System	Mechanical Ventilation	↓: GPX4, SOD2 ↑: 4-HNE	Zhou et al., 2020[74]
	dexmedetomidine	↑: GSH, GPX4 ↓: TNF-α, LPO, MDA, LDH, COX2, ACSL4	Tao et al., 2022[75]

* ↑ stands for being up-regulated; ↓ stands for being down-regulated.

## Author contribution statement

All authors listed have significantly contributed to the development and the writing of this article.

## Funding statement

This research did not receive any specific grant from funding agencies in the public, commercial, or not-for-profit sectors.

## Additional information

No additional information is available for this paper.

## Data availability statement

No data was used for the research described in the article.

## Declaration of competing interest

The authors declare that they have no known competing financial interests or personal relationships that could have appeared to influence the work reported in this paper.
